# North-Eastern Hospital for Children

**Published:** 1890-07-12

**Authors:** Henry A. Isaacs, R. C. Bedford, J. Gurney Barclay

**Affiliations:** Lord Mayor; Bishop Suffragan for East London; President


### Editor's Letter-Box.

[Our Correspondents are reminded that prolixity is a great bar to publication, and that brevity of style and conciseness of statement greatly facilitate early insertion.]

### NORTH-EASTERN HOSPITAL FOR CHILDREN, HACKNEY ROAD, N.E.

Will you allow us the privilege of a short space in your columns for an urgent appeal on behalf of the North-Eastern Hospital for Children?

At a meeting held in the Mansion House the following resolutions were adopted:—

1. That this meeting considers the North-Eastern Hospital for Children well deserving of public recognition and support, on account of the good and efficient work it has done for many years and is now doing in a very densely-populated and poor quarter of East London.

2. That an appeal be made to the public for the sum of £3,000.

This sum is required to free the hospital from debt, and to meet the current expenses of the charity.

During the past year 531 in-patients have been received and 14,550 out-patients relieved.

There is a dense and poor population of about three-quarters of a million within a radius of one mile of the hospital.

The total expenditure last year was £5,089.

We are of opinion that few charities can show a better account of good work done at comparatively a very small cost. This appeal will surely commend itself to all who love children, and we trust the Committee of Management may be relieved from all fear of financial embarrassment and the charity set free to continue to care for the multitude of suffering children in the streets and courts of this part of the metropolis. Contributions may be sent to the honorary Treasurer, Mr. J. Lister Godlee, 3, New Square, Lincoln's Inn, W.C., or to the Secretary, Mr. Alfred Nixon, 27, Clement's Lane, E.C., who will gladly receive and acknowledge either donations or subscriptions.

HENRY A. ISAACS, Lord Mayor.

R. C. BEDFORD, Bishop Suffragan for East London.

J. GURNEY BARCLAY, President.

